# Supplementary material for: Abdominal Obesity-Related Disturbance of Insulin Sensitivity Is Associated with CD8+ EMRA Cells in the Elderly
Source: Cells. 2021 Apr 23;10(5):998. doi: 10.3390/cells10050998 (PMC8146929; doi:10.3390/cells10050998)
Supplement: Supplementary file 1 [file cells-10-00998-s001.zip › cells-1172886-supplementary.pdf]

Table S1. T cell subpopulations (mean ± SD) of all participants.

T cell subpopulations	
CD4/CD8 ratio	3.96 ± 2.45
**CD4+ T cells [% CD3+]**	70.78 ± 9.86
naïve cells [% CD4+]	41.35 ± 14.49
Central Memory cells [% CD4+]	39.98 ± 9.31
Effector Memory cells [% CD4+]	17.12 ± 8.19
EMRA cells [% CD4+]	1.71 ± 1.41
**CD8+ T cells [% CD3+]**	22.33 ± 8.94
naïve cells [% CD8+]	27.14 ± 16.38
Central Memory cells [% CD8+]	3.39 ± 2.45
Effector Memory cells [% CD8+]	43.77 ± 14.63
EMRA cells [% CD8+]	25.76 ± 15.91
**Regulatory T cells [% CD3+]**	6.05 ± 1.65

Table S2. Cytokine and adipokine levels (mean ± SD) of all participants.

Cytokines/adipokines	
CX3CL1/Fractalkine [pg/ml]	4116.9 ± 249.5
IL-1β [pg/ml]	2.34 ± 2.40
IL-1ra [pg/ml]	360.20 ± 338.36
IL-6 [pg/ml]	1.22 ± 1.38
IL-10 [pg/ml]	0.48 ± 0.42
IL-15 [pg/ml]	2.30 ± 2.50
TNF-α [pg/ml]	2.56 ± 1.59
Adiponectin [pg/ml]	39.98 ± 9.31
Resitin [pg/ml]	17.12 ± 8.19

Table S3. Tryptophan metabolites (mean ± SD) of all participants.

Tryptophan metabolites	
Tryptophan (TRP) [μmol/L]	50.23 ± 15.11
Kynurenine (KYN) [μmol/L]	1.36 ± 0.38
Quinolinic acid (QA) [μmol/L]	0.42 ± 0.11
Kynurenic acid (KYNA) [μmol/L]	0.033 ± 0.013
KYN/TRP	0.028 ± 0.009
QA/KYN	0.323 ± 0.075
KYNA/KYN	0.025 ± 0.007
QA/KA	14.029 ± 6.674
